# Thrombocytopenia in critically ill trauma patients is associated with the pattern and duration of postinjury organ dysfunction

**DOI:** 10.1016/j.rpth.2025.102890

**Published:** 2025-05-17

**Authors:** Andrea Rossetto, Simon Kerner, Ella Ykema, Harriet E. Allan, Paul C. Armstrong, Elaine Cole, Paul Vulliamy

**Affiliations:** 1 Centre for Trauma Sciences, Blizard Institute, Queen Mary University of London, London, UK; 2 Trauma Service, Barts Health National Health Service Trust, London, UK; 3 Centre for Immunobiology, Blizard Institute, Queen Mary University of London, London, UK

**Keywords:** critical care, life support care, multiple organ failure, thrombocytopenia, trauma

## Abstract

**Background:**

Although significant thrombocytopenia is not a common feature of trauma patients in the first hours after injury, little is known about how severe trauma affects platelet count trajectories beyond the initial resuscitation phase and whether changes in platelet count are related to clinical outcomes such as multiple organ dysfunction syndrome and mortality.

**Objectives:**

To define the incidence, severity, and clinical significance of postinjury thrombocytopenia during critical care admission.

**Methods:**

Trauma patients enrolled in a perpetual cohort study at a single level 1 trauma center between 2014 and 2023 who required critical care admission were included. Thrombocytopenia was classified as mild (100-149 × 10^9^/L), moderate (50-99 × 10^9^/L), and severe (<50 × 10^9^/L). Multivariable regression analyses were used to investigate the drivers of thrombocytopenia and its association with outcomes of organ dysfunction, organ support, and mortality.

**Results:**

Among 803 trauma patients investigated, mild, moderate, and severe thrombocytopenia occurred in 285 (35%), 290 (36%), and 51 (6%), respectively, with the nadir mostly between 48 and 72 hours of their critical care stay. Age, injury severity, shock, admission coagulopathy, and total fluid administration within the first 24 hours were all independently associated with the development of moderate-severe thrombocytopenia. Thrombocytopenia of any severity was independently associated with renal and hepatic dysfunction, but not with cardiorespiratory dysfunction or mortality. Severe thrombocytopenia was also independently associated with prolonged need for organ support (odds ratio, 2.83; 95% CI, 1.07-7.45; *P* = .04).

**Conclusion:**

Thrombocytopenia is common in injured patients admitted to critical care, and severe forms are independently associated with a higher incidence of organ dysfunction and need for organ support.

## Essentials

• Little is known about how trauma affects platelet count trajectories after initial resuscitation.
• Trauma patients enrolled in a perpetual cohort study at a level 1 trauma center from 2014 to 2023.
• Thrombocytopenia is common in trauma patients admitted to critical care in the days after injury.
• Severe thrombocytopenia is related to higher incidence of organ dysfunction and organ support.

## 1 Introduction

Major trauma results in dramatic changes in platelet behavior, including decreased aggregatory function [1,2], loss of response to collagen [3,4], increased surface markers of activation [3,4], and procoagulant transformation [5]. Most previous studies of postinjury platelet biology have focused on how platelets contribute to trauma-induced coagulopathy and have largely examined changes in the acute phase after injury [1,6]. Beyond their roles in hemostasis, platelets are also important contributors to acute inflammatory responses and host defense [7,8]. Trauma patients who survive the immediate postinjury period are at high risk of multiple organ dysfunction syndrome (MODS), which accounts for much of the critical care morbidity and mortality [9,10], but the roles of platelets in the development of posttrauma MODS have not yet been defined in detail.

In addition to early alterations in platelet function after injury, changes in platelet counts during the initial hours after major trauma appear to be clinically important. Although significant thrombocytopenia is not usually a feature of trauma-induced coagulopathy, acute (<24 hours) reductions in platelet count, even within the normal range, are associated with worse outcomes [6,11]. Beyond 24 hours, less is known about how severe trauma affects platelet count trajectories, and how any changes are related to clinical outcomes. In a recent large multicenter study of mixed intensive care unit (ICU) patients, thrombocytopenia was present in over a third of patients and was associated with increased mortality and resource utilization [12]. However, this study only included a small number of injured patients and did not specifically investigate this subgroup. We have recently shown that platelet production dynamics are altered after major trauma, with sustained increases in circulating immature platelets in patients who develop organ failure and thrombosis [13]. To date, an in-depth study of platelet count trajectories after severe injury, and in particular, the significance of thrombocytopenia during posttraumatic critical illness, has not yet been reported.

Our primary objective was to define the incidence, severity, and clinical significance of postinjury thrombocytopenia during critical care admission. We hypothesized that thrombocytopenia would be prevalent and that more severe thrombocytopenia would be associated with higher rates of organ dysfunction, need for organ support, and mortality.

## 2 Methods

### 2.1 Study design

Patients were recruited into the perpetual prospective observational cohort study Activation of Coagulation and Inflammation in Trauma (ACIT-II) study (research etichs commitee reference 07/Q0603/29, ISRCTN12962642). Adult patients requiring activation of the trauma team are screened for inclusion. Exclusion criteria are time from injury >2 hours, administration of >2000 mL fluid prehospital, and burns >5% body surface area. For the purpose of this study, we included all patients admitted to critical care (high dependency unit or ICU) over a 10-year period between 2014 and 2023 and who had ≥1 platelet count measurement recorded during the critical care stay. Consent procedures for the ACIT-II study have been described in detail previously [14].

### 2.2 Study procedures

Demographic and injury data were recorded prospectively by a trained member of the research team. Participants were followed up daily from the date of injury (designated as day 0) until day 28 after injury, until death, or discharge, if this occurred sooner. Variables collected included: daily Sequential Organ Failure Assessment (SOFA) component scores during critical care stay (including platelet count measurements recorded as the hematology component); need for vasopressor support, invasive ventilation, and renal replacement therapy (RRT); incidence and type of venous thromboembolic events; and mortality.

Blood samples for research purposes were drawn at admission, at 24 hours, and at 72 hours. The immature platelet fraction was measured at these time points in a subgroup of the main cohort as part of a separate study using a Sysmex XN-series analyzer according to standard operating procedures, and immature platelet count was calculated as previously described [13] Rotational thromboelastometry was performed in citrated whole blood using a Delta instrument according to the manufacturer’s instructions. Base deficit and lactate were measured by point-of-care blood gas analysis.

### 2.3 Outcomes

Our primary outcome was organ dysfunction and failure, as measured by the SOFA score. To exclude the confounding effect of the hematological component (platelet count), we modified the SOFA score as previously described (mSOFA) [15,16], and considered both the overall mSOFA score and the individual component scores in our analyses. We did not specifically evaluate the central nervous component due to the challenges of accurate daily Glasgow Coma Score measurement in the presence of critical care sedation [17]. Secondary outcomes were ventilator days, vasopressor days, need for RRT, composite time to complete organ failure recovery (CTCOFR) [18,19], prolonged organ support, in-hospital mortality, and venous thromboembolism.

### 2.4 Definitions

Thrombocytopenia was defined as a platelet count of <150 × 10^9^/L and further classified as mild (100-149 × 10^9^/L), moderate (50-99 × 10^9^/L), and severe (<50 × 10^9^/L) thrombocytopenia according to previously published definitions [12]. Patients were subdivided into 4 groups using these cutoffs according to the lowest count recorded during the critical care stay. Admission injury severity score (ISS) and base deficit were used as markers of injury burden and shock, respectively. Coagulopathy was defined as clot amplitude <40mm on tissue-factor activated rotational thromboelastometry [20]. Massive hemorrhage was defined as the requirement for ≥10 units of red blood cells in the first 24 hours. Organ dysfunction and failure in individual organ systems were defined as SOFA component scores of >0 and >2, respectively [21]. mSOFA was defined as the presence of at least 2 SOFA components with a score >2, with the exclusion of the hematological component [15,16]. CTCOFR was defined as the total number of days before mechanical ventilation, vasopressor therapy, and RRT were all discontinued, with a score of 0 for those patients who did not require any of these organ support interventions, a score of 15 for those who required any of these interventions for >14 days, and a score of 16 for those who died at any time during the study period [18,19]. Prolonged organ dysfunction was defined as a CTCOFR of >7, in line with existing definitions [22].

### 2.5 Statistical analysis

Analysis was performed using Prism v10.4.1 (GraphPad Software Inc) and R v4.1.3 (R Core Team). Continuous data are reported as median with IQR and were compared using the Kruskal–Wallis test with Dunn posttest correction for multiple comparisons. Categorical data are reported as number and percentage and were compared using Fisher exact test or chi-squared test with Bonferroni correction. No multiple imputation for missing data was performed. A multinomial logistic regression analysis was used to investigate the factors associated with the occurrence of mild, moderate, and severe thrombocytopenia. We included the following predictor variables: demographics (sex, as recorded on the electronic medical records, and age), ISS, shock as measured by base deficit, coagulopathy, and total fluids and blood products administered in the first 24 hours. Multivariable linear and logistic regressions were then used to investigate the association between thrombocytopenia categories and clinical outcomes of organ dysfunction/failure, organ support requirements, and mortality, adjusting for the abovementioned predictor variables and the mechanism of injury. All variables were included in the multivariable regressions independent of their statistical significance in the univariable analysis [23]. Results are reported as odds ratios (ORs) for categorical variables or coefficients for continuous variables with 95% CIs. A 2-tailed *P* value < .05 was considered significant throughout.

## 3 Results

### 3.1 Patient characteristics

Of 1800 trauma patients enrolled into the ACIT-II study between 2014 and 2023, the cohort included in this study comprised 803 patients admitted to critical care and who had ≥1 measurement of platelet count during the critical care stay. The majority were male (651/803, 81%) and had sustained a blunt mechanism of injury (622/803, 77%) (Table 1). Patients were severely injured, with a median ISS of 26 (17-38), a median critical care length of stay of 8 (4-17) days, and an overall in-hospital mortality of 16% (132/803).

**Table 1:** Characteristics of the study cohort stratified by the severity of thrombocytopenia in critical care.

Variables	Overall (*N* = 803)	No thrombocytopenia (*n* = 177)	Mild thrombocytopenia (*n* = 285)	Moderate thrombocytopenia (*n* = 290)	Severe thrombocytopenia (*n* = 51)
**Admission characteristics**					
Sex, male	651 (81.1)	145 (81.9)	229 (80.4)	240 (82.8)	37 (72.5)
Age, y	37 (25-55)	37 (26-53)	38 (26-58)	35 (24-53)	36 (26-53)
Glasgow coma score	11 (5-15)	11 (6-15)	11 (5-15)	11 (6-15)	10 (3-14)
Base deficit, mEq/L	4 (1-9)	2 (0-5)	3 (1-7)^a^	6 (3-11)^a^^,^^b^	10 (5-18)^a^^,^^b^^,^^c^
SBP, mmHg	117 (92-139)	127 (106-146)	119 (95-140)	110 (89-134)^a^^,^^b^	97 (75-126)^a^^,^^b^
INR >1.2	154 (23.7)	12 (8.3)	47 (20.1)^a^	81 (34.3)^a^^,^^b^	14 (38.9)^a^
EXTEM A5, mm	40 (34-45)	45 (40-49)	41 (36-46)^a^	36 (31-42)^a^^,^^b^	32 (22-41)^a^^,^^b^
**Injury characteristics**					
Mechanism of injury	622 (77.5)	138 (78.0)	220 (77.2)	223 (76.9)	41 (80.4)
Injury severity score	26 (17-38)	24 (16-30)	26 (17-35)^a^	29 (19-41)^a^	32 (25-43)^a^^,^^b^
AIS Head/Neck ≥3	396 (50.2)	94 (54.7)	153 (54.3)	126 (44.1)	23 (46.9)
AIS Face ≥3	32 (4.1)	6 (3.5)	10 (3.6)	13 (4.5)	3 (6.1)
AIS Thorax ≥3	423 (53.2)	85 (48.6)	152 (53.7)	165 (57.5)	21 (42.0)
AIS Abdomen ≥3	185 (23.5)	27 (15.5)	55 (19.7)	87 (30.6)^a^^,^^b^	16 (32.0)
AIS Extremity ≥3	280 (35.4)	27 (15.7)	79 (28.0)^a^	145 (50.3)^a^^,^^b^	29 (58.0)^a^^,^^b^
AIS External ≥3	19 (2.5)	1 (0.6)	10 (3.7)	5 (1.8)	3 (6.7)
**Fluids and BP in first 24 h**					
Crystalloids, L	3.00 (2.00-4.20)	2.03 (1.32-3.38)	3.00 (2.00-4.01)^a^	3.18 (2.20-4.5)^a^^,^^b^	3.40 (2.50-5.00)^a^
Red blood cells, units	2 (0-6)	0 (0-2)	2 (0-4)^a^	4 (2-8)^a^^,^^b^	9 (5-17)^a^^,^^b^^,^^c^
Massive hemorrhage	87 (11.1)	3 (1.7)	9 (3.2)	52 (18.1)^a^^,^^b^	23 (46.9)^a^^,^^b^^,^^c^
Fresh frozen place, units	1 (0-5)	0 (0-0)	0 (0-4)^a^	4 (0-8)^a^^,^^b^	8 (4-14)^a^^,^^b^^,^^c^
Platelets, pools	0 (0-1)	0 (0-0)	0 (0-1)^a^	1 (0-2)^a^^,^^b^	1 (1-3)^a^^,^^b^^,^^c^
Cryoprecipitate, pools	0 (0-2)	0 (0-0)	0 (0-1)^a^	2 (0-3)^a^^,^^b^	3 (1-6)^a^^,^^b^^,^^c^

Data presented as median (IQR) or count (percentage).

AIS, abbreviated injury severity score; BP, blood products; EXTEM, tissue-factor activated rotational thromboelastometry; INR, international normalized ratio; SBP, systolic blood pressure.

a *P* < .05 when compared with no thrombocytopenia.

b *P* < .05 when compared with mild thrombocytopenia.

c *P* < .05 when compared with moderate thrombocytopenia.

### 3.2 Platelet count trajectories after injury

**Figure 1:**
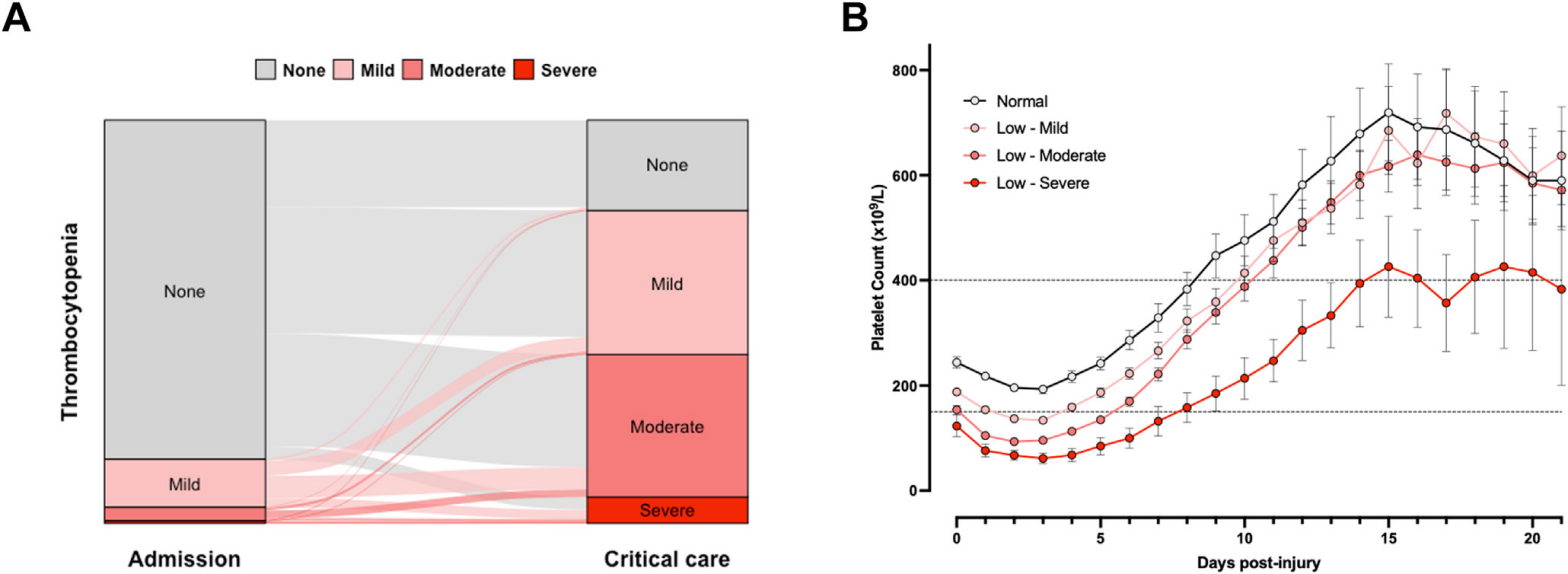
Platelet count trajectories after injury. (A) Sankey plot depicting thrombocytopenia patterns on arrival in the emergency department (left column) and during critical care stay (right column). None, no thrombocytopenia; mild (100-149 × 10^9^/L), moderate (50-99 × 10^9^/L), and severe (<50 × 10^9^/L). (B) Platelet counts over time in patients grouped by lowest platelet count during critical care stay. Mean with 95%CIs. Dashed lines denote normal range.

On arrival in the emergency department, thrombocytopenia was present in 119 of 803 patients (15%), most of whom had mild reductions in platelet count only (127 × 10^9^/L; IQR, 100-139). During the critical care stay, the incidence of thrombocytopenia rose to 78% (626/803), with markedly higher rates of moderate (36.1% vs 3.1%, *P* < .001) and severe (6.4% vs 0.6%, *P* < .001) thrombocytopenia compared with admission (Figure 1A). Overall, mild, moderate, and severe thrombocytopenia occurred in 285 (35%), 290 (36%), and 51 (6%) patients, respectively, during the critical care stay. Longitudinal platelet count trajectories were similar in these subgroups, with an initial decline in platelet count, reaching a nadir around 2 days (IQR, 1-3) after injury with minor differences according to the presence or severity of thrombocytopenia (Figure 1B). In patients with mild and moderate thrombocytopenia, platelet counts recovered by day 7 before rising to supranormal levels by day 14 and beginning to return to normal thereafter. Patients with severe thrombocytopenia showed a similar trajectory, but with a slower recovery, lower peak, and persistently lower count throughout the critical care stay.

### 3.3 Factors associated with postinjury thrombocytopenia

We next examined the clinical characteristics of patients stratified by severity of thrombocytopenia during their critical care admission. Patients with moderate and severe thrombocytopenia were more severely injured, shocked, and coagulopathic on arrival compared to those with mild and no thrombocytopenia (Table 1). However, there were no differences in mechanism of injury, patient age, or incidence of traumatic brain injury across the 4 groups. Volumes of blood products (including platelet transfusions) and crystalloid administered in the first 24 hours after injury were higher with increasing degree of thrombocytopenia. However, the hematocrit and hemoglobin concentrations over the first 72 hours were similar irrespective of the degree of thrombocytopenia (Supplementary Figure S1). On multivariable analysis, we found that older age, injury severity, degree of shock, admission coagulopathy, and total fluid administration within the first 24 hours were all independently associated with development of both moderate and severe thrombocytopenia (Table 2).

**Table 2:** Multinomial logistic regression analysis for severity of thrombocytopenia in critical care.

Variables	Severity of thrombocytopenia	OR (95% CI)	*P*	Adj. OR (95% CI)	Adj. *P*
Sex, male	Mild	0.80 (0.47-1.36)	.40	0.95 (0.54-1.66)	.85
	Moderate	0.90 (0.53-1.54)	.70	1.04 (0.56-1.92)	.91
	Severe	0.40 (0.18-0.87)	.02	0.44 (0.18-1.08)	.07
Age, y	Mild	1.01 (1.00-1.02)	.12	1.02 (1.00-1.03)	.007
	Moderate	1.00 (0.99-1.01)	.72	1.02 (1.00-1.03)	.02
	Severe	1.00 (0.98-1.02)	.97	1.02 (1.00-1.05)	.04
Injury severity score	Mild	1.02 (1.00-1.04)	.02	1.01 (1.00-1.03)	.11
	Moderate	1.04 (1.03-1.06)	<.001	1.02 (1.01-1.04)	.01
	Severe	1.05 (1.03-1.08)	<.001	1.03 (1.00-1.06)	.03
Base deficit, mEq/L	Mild	1.06 (1.01-1.11)	.02	1.02 (0.97-1.07)	.48
	Moderate	1.15 (1.10-1.20)	<.001	1.05 (1.00-1.10)	.05
	Severe	1.24 (1.17-1.30)	<.001	1.11 (1.04-1.18)	.001
EXTEM A5 <40 mm	Mild	2.10 (1.32-3.33)	.002	1.84 (1.14-2.98)	.01
	Moderate	6.99 (4.40-11.1)	<.001	4.60 (2.77-7.65)	<.001
	Severe	7.61 (3.57-16.2)	<.001	2.99 (1.23-7.29)	.02
Total fluids and BP in first 24 h, L	Mild	1.22 (1.12-1.33)	<.001	1.21 (1.10-1.33)	<.001
	Moderate	1.51 (1.38-1.64)	<.001	1.42 (1.29-1.56)	<.001
	Severe	1.70 (1.53-1.87)	<.001	1.57 (1.40-1.75)	<.001

*R*^2^ = 0.08/0.32/0.51. Cases per variable = 25.0/25.0/7.0.

Adj., adjusted; BP, blood products; EXTEM, tissue-factor activated rotational thromboelastometry; OR, odds ratio.

We also examined immature platelet metrics in a subgroup of 92 patients (no thrombocytopenia, *n* = 19; mild, *n* = 29; moderate, *n* = 39; severe, *n* = 5); characteristics of this subgroup are reported in Supplementary Table S1. We found no statistically significant differences in either immature platelet fraction or immature platelet count at admission, 24 hours, or 72 hours in patients stratified according to thrombocytopenia severity (Supplementary Figure S2).

### 3.4 Association between thrombocytopenia severity and clinical outcomes

**Figure 2:**
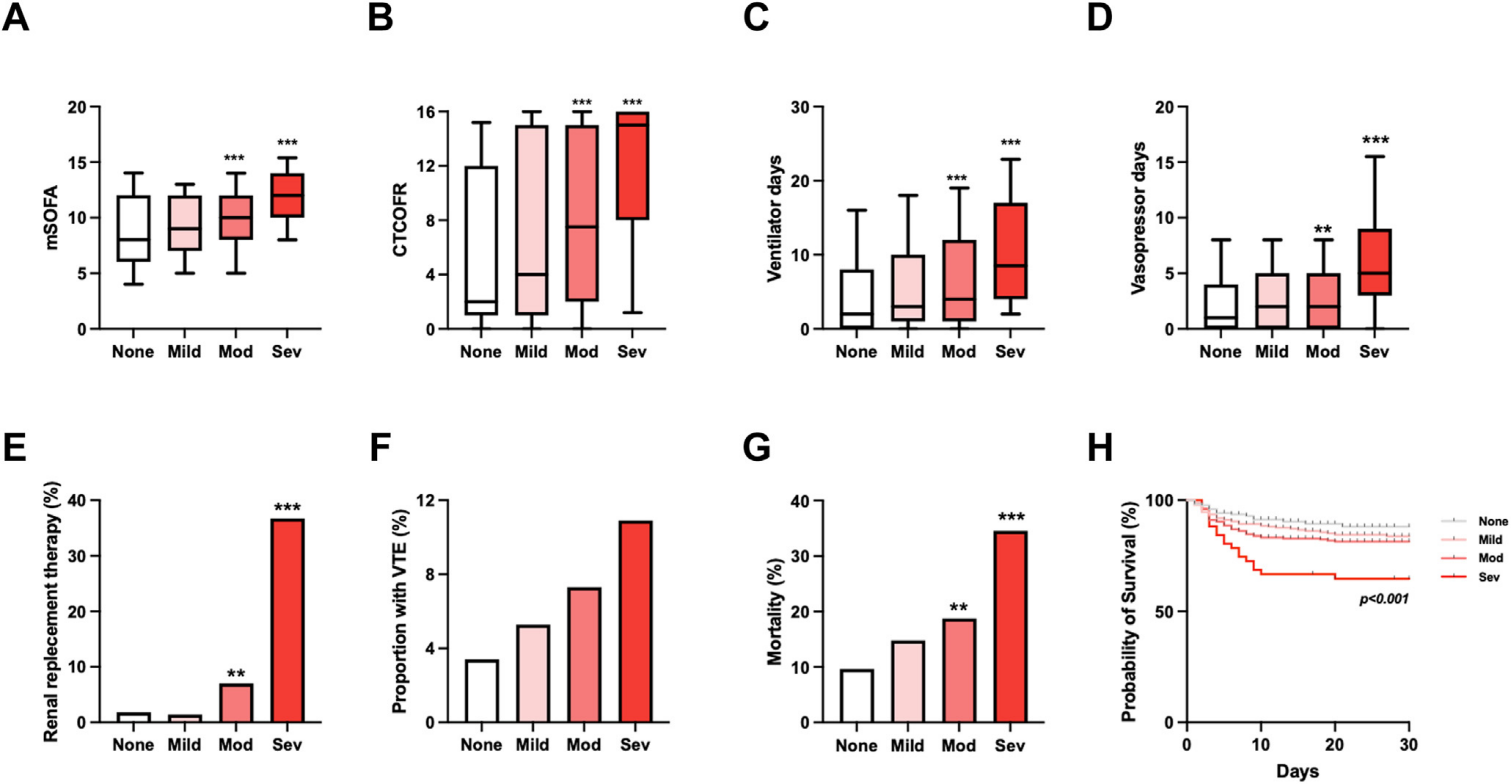
Association between severity of thrombocytopenia with clinical outcomes. (A) Modified Sequential Organ Failure Assessment (mSOFA) score, calculated as sum of all individual component scores except platelet count. (B) Composite time to complete organ failure (CTCOFR). (C) Ventilator days. (D) Days requiring vasopressor support. (E) Renal replacement therapy use. (F) Venous thromboembolism (VTE). (G) In-hospital mortality. (H) Kaplan–Meier curves for survival. *P* value derived using log-rank test. Mod, moderate; Sev, severe. ∗∗*P* < .01; ∗∗∗*P* < .001 vs non–thrombocytopenia, Kruskal–Wallis test with Dunn posttest correction. Box–whisker plots depict 10^th^ to 90th percentiles.

Patients with moderate and severe thrombocytopenia had significantly higher mSOFA (Figure 2A) and CTCOFR scores (Figure 2B) than those without thrombocytopenia. SOFA component scores for cardiovascular, renal, and hepatic dysfunction were also consistently higher throughout critical care stay in patients with severe thrombocytopenia, while respiratory scores were similar across the 4 groups (Supplementary Figure S3). Organ support requirements were significantly higher in patients with moderate and severe thrombocytopenia, with higher ventilator days (Figure 2C), vasopressor days (Figure 2D), and proportions requiring RRT (Figure 2E). Rates of venous thromboembolism rose in a linear manner with increasing severity of thrombocytopenia, although this trend did not reach statistical significance (Figure 2F). Overall mortality was significantly higher in patients with moderate and severe thrombocytopenia than in those with no thrombocytopenia (Figure 2G, H).

**Figure 3:**
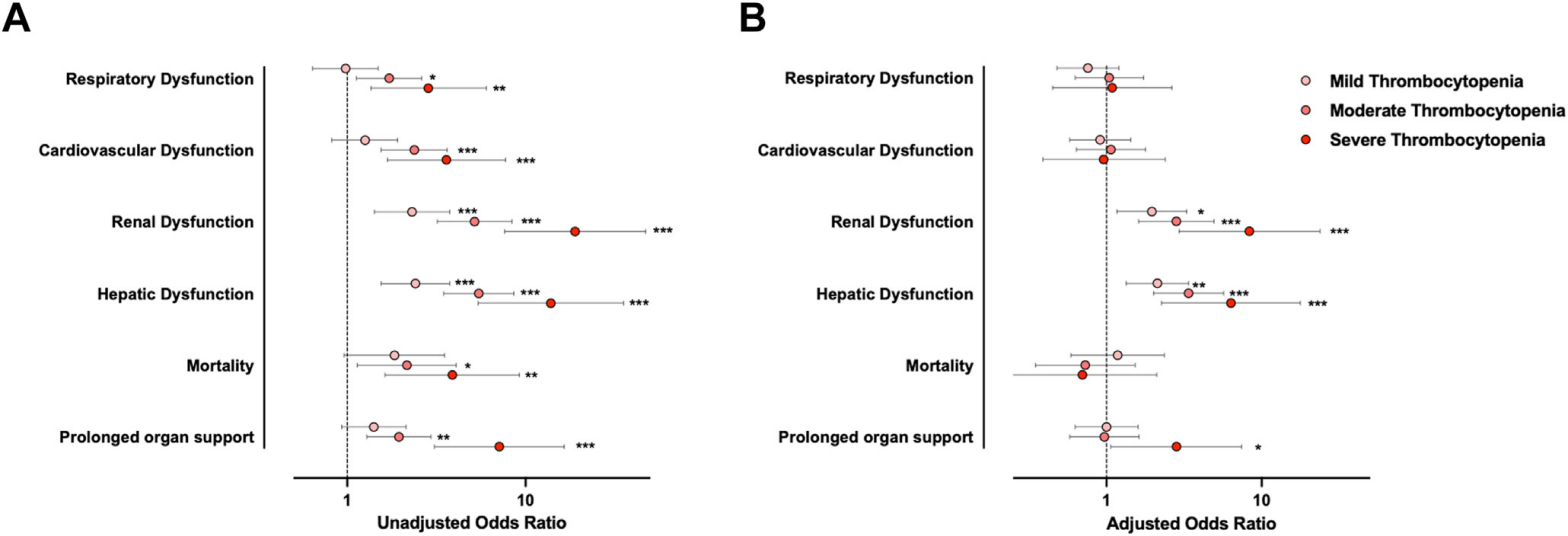
Multivariable regression analysis for organ dysfunction, mortality, and prolonged organ support. (A) Univariable models. (B) Multivariable models after adjusting for age, sex, injury severity score, mechanism of injury, admission base deficit, presence of coagulopathy, and total fluid volumes administered within 24 hours. Full details of all models are reported in Supplementary Table S2. Organ dysfunction defined as a SOFA component score >0 for the cardiovascular, renal, and hepatic components and >2 for the respiratory component. Prolonged organ support defined as a composite time to complete organ failure recovery (CTCOFR) score >7. Circles are odds ratios relative to no thrombocytopenia. Error bars represent 95% CIs. ∗*P* < .05; ∗∗*P* < .01; ∗∗∗*P* < .001.

To further investigate the relationship between thrombocytopenia severity and outcome, we constructed a series of regression models. On univariable analysis, both moderate and severe thrombocytopenia were associated with respiratory, cardiovascular, renal, and hepatic dysfunction, as well as higher odds of prolonged organ support and mortality (Figure 3A). On multivariable analysis, mild, moderate, and severe thrombocytopenia remained independently associated with renal (mild: OR, 1.96; 95% CI, 1.17-3.29; *P* = .01; moderate: OR, 2.82; 95% CI, 1.61-4.93; *P* < .001; severe: OR, 8.33; 95% CI, 2.93-23.6; *P* ≤ .001), and hepatic dysfunction (mild: OR, 2.13; 95% CI, 1.34-3.39; *P* = .001; moderate: OR, 3.38; 95% CI, 2.01-5.69; *P* < .001; severe: OR, 6.34; 95% CI, 2.26-17.8; *P* ≤ .001) but not with cardiorespiratory dysfunction or mortality after adjustment for relevant confounders (Figure 3B and Supplementary Table S2). Severe thrombocytopenia was also independently associated with prolonged need for organ support (OR, 2.83; 95% CI, 1.07-7.45; *P* = .04), CTCOFR (coefficient, 2.27; 95% CI, 0.11-4.44; *P* = .04), ventilator days (coefficient, 0.85; 95% CI, 0.14-1.55; *P* = .02), vasopressor days (coefficient, 0.70; 95% CI, 0.21-1.19; *P* = .005), and need for RRT (OR, 9.22; 95% CI, 1.42-59.8; *P* = .02) on multivariable analysis (Supplementary Table S3).

## 4 Discussion

In this study of >800 critically ill major trauma patients, we report the clinical relevance and drivers of postinjury thrombocytopenia. Our central findings are that some degree of thrombocytopenia is extremely common in critical care trauma patients; that moderate-severe depletion of platelets is associated with both endogenous and iatrogenic factors; and that severe thrombocytopenia is independently associated with a higher incidence of organ dysfunction and need for organ support, but not with mortality. These findings suggest a process of increased platelet destruction, sequestration, dilution, and/or consumption after injury that is associated with progression to organ failure. The mechanisms involved in this process are not clear and warrant further mechanistic investigation.

More than three-quarters of injured patients in this study developed some degree of thrombocytopenia, a significantly higher proportion than in previous studies of critically ill patients. In the recent thrombocytopenia and platelet transfuions in ICU patients study [12], the overall incidence of thrombocytopenia in a mixed ICU population (primarily with cardiorespiratory failure and/or sepsis) was 43%, and in a large study of neonatal patients postcardiac surgery, the incidence was 34% [24]. The dynamics of thrombocytopenia in these populations were also different than the postinjury response, with postcardiac surgery patients recovering steadily from an earlier nadir on postoperative day 1, and the mixed critically ill population in thrombocytopenia and platelet transfusions in ICU patients having higher rates of admission thrombocytopenia than trauma patients and a slightly later overall nadir on day 3. Collectively, this suggests that although platelet depletion appears to be a common feature in multiple acutely ill patient populations, the incidence and potential underlying mechanisms vary across disease states.

We found that postinjury thrombocytopenia occurs due to a combination of age, injury-related factors, and the dilutional effect of fluid resuscitation given in the immediate postinjury period. Both injury load and degree of shock were independently associated with the development of moderate-severe thrombocytopenia. Platelet depletion in critical illness is often variously attributed to consumption, sequestration, and/or enhanced clearance, but direct evidence for these processes in trauma patients is lacking. However, given our observation here that early immature platelet metrics do not correlate with thrombocytopenia, it appears that peripheral depletion of platelets rather than bone marrow failure is primarily responsible. Both tissue injury and systemic hypoperfusion lead to release of damage-associated molecular patterns (DAMPs) [25], many of which are known to directly induce thrombocytopenia [26] and modulate platelet activity [5,27,28]. We postulate that DAMP-mediated platelet depletion could represent a central mechanism leading to postinjury thrombocytopenia; however, further work is required to delineate the specific pathways involved, and in particular, to interrogate the temporal relationship between DAMP release and dynamic changes in platelet number and function, as well as define the contribution of individual DAMPs. Another important area for further study is the functional properties and phenotype of platelets at later timepoints after injury, which has not received the same focus as studies focusing on the immediate postinjury period. Previous work has shown a persistent impairment in *ex vivo* responsiveness extending for several days after injury [1,29], but detailed profiling of platelet phenotype—and determining how this relates to platelet count dynamics—has not yet been performed.

In contrast to the aforementioned studies of thrombocytopenia in other patient populations [12,24], we did not find an independent association between thrombocytopenia and mortality. However, we found that severely low platelet counts were associated with prolonged need for organ support and that thrombocytopenia overall was associated with liver and renal (although not cardiorespiratory) dysfunction. Severe thrombocytopenia in septic patients has been shown to result in specific perturbations in immune function, including increased cytokine levels, endothelial cell activation, and impaired vascular integrity [15]. Consistent with this, our findings suggest that marked depletion of platelets after injury confers a greater risk of more severe and more prolonged organ failure, with implications for resource utilization and patient morbidity. The immunological mechanisms that may be involved in the trauma setting remain to be elucidated.

This study is limited by its single center observational design, which limits generalizability and potential for causal inference. As our focus in this study was on platelet count dynamics, we did not perform functional studies on platelets across different time points or measure levels of candidate DAMPs that may contribute to thrombocytopenia, both of which are important areas for further study. In addition, data were lacking on fluid volumes administered beyond 24 hours, incidence of nosocomial infection, and immature platelet metrics for the full cohort. We did not specifically evaluate the effect of platelet transfusions given after resuscitation on platelet counts, and this remains an important area for further study. Finally, because we used the SOFA score to quantify organ dysfunction, we were not able to directly examine the relationship between platelet counts and MODS risk because the complete SOFA score calculation includes platelet count. To overcome this limitation, we focused on organ support, which represents a more direct measure of the resource utilization associated with organ dysfunction.

In summary, this study provides a detailed insight into the factors associated with postinjury thrombocytopenia and its clinical relevance. In contrast with other patient populations, the severity of thrombocytopenia in critical care is not independently associated with mortality but is linked to the pattern of organ dysfunction and the overall requirement for organ support. The main factors associated with development of thrombocytopenia are age, injury burden, degree of hypoperfusion at admission, and fluid resuscitation volumes. Further work is required to interrogate the underlying mechanisms responsible for postinjury thrombocytopenia and the relationship between platelet depletion and altered immunological responses.
