# Supplementary material for: Thrombocytopenia in critically ill trauma patients is associated with the pattern and duration of postinjury organ dysfunction
Source: Res Pract Thromb Haemost. 2025 May 17;9(4):102890. doi: 10.1016/j.rpth.2025.102890 (PMC12178710; doi:10.1016/j.rpth.2025.102890)
Supplement: Supplementary Material [file mmc1.docx]

# ­Supplementary file content:

- **Supplementary Table S1.** Characteristics of the immature platelet fraction (IPF) cohort stratified by severity of thrombocytopenia in critical care.
- **Supplementary Table S2.** Multivariable logistic regression analysis for mortality, organ dysfunction/failure and organ failure resolution.
- **Supplementary Table S3.** Multivariable linear regression analysis for organ failure resolution and organ support.
- **Supplementary Figure S1.** Haematocrit and haemoglobin levels over time stratified by severity of thrombocytopenia.
- **Supplementary Figure S2.** I immature platelet fraction (IPF) and immature platelet count (IPC) levels over time stratified by severity of thrombocytopenia.
- **Supplementary Figure S3.** Organ injury score trajectories stratified by severity of thrombocytopenia.

| **Supplementary Table S1. Characteristics of the IPF cohort stratified by the severity of thrombocytopenia in critical care** | | | | | |
| --- | --- | --- | --- | --- | --- |
|  | **Overall** | **No Thrombocytopenia** | **Mild Thrombocytopenia** | **Moderate Thrombocytopenia** | **Severe Thrombocytopenia** |
|  | (n = 92) | (n = 19) | (n = 29) | (n = 39) | (n = 5) |
| **Admission characteristics** |  |  |  |  |  |
| Sex, male | 75 (81.5) | 15 (78.9) | 22 (75.9) | 33 (84.6) | 5 (100.0) |
| Age, years | 35 (22-53) | 33 (23-44) | 32 (21-56) | 40 (23-53) | 35 (25-41) |
| Glasgow coma score | 12 (4-15) | 14 (7-15) | 14 (8-15) | 8 (3-14) | 3 (3-3) |
| Base deficit, mEq/L | 5 (2-9) | 3 (0-7) | 2 (1-7) | 7 (4-15) | 5 (2-17) |
| SBP, mmHg | 114 (98-131) | 117 (96-139) | 118 (107-127) | 111 (101-131) | 93 (59-127) |
| INR >1.2 | 21 (29.2) | 3 (17.6) | 6 (25.0) | 11 (36.7) | 1 (100.0) |
| EXTEM A5, mm | 37 (30-42) | 39 (36-45) | 39 (32-44) | 35 (29-39) | 19 (12-31) |
| **Injury characteristics** |  |  |  |  |  |
| Mechanism of injury | 65 (70.7) | 13 (68.4) | 17 (58.6) | 32 (82.1) | 3 (60.0) |
| Injury severity score | 29 (21-38) | 21 (13-27) | 25 (17-33) | 36 (25-45) | 30 (30-35) |
| AIS Head/Neck ≥ 3 | 40 (45.5) | 7 (46.7) | 11 (37.9) | 19 (48.7) | 3 (60.0) |
| AIS Face ≥ 3 | 4 (4.5) | 1 (6.3) | 1 (3.4) | 2 (5.1) | 0 (0.0) |
| AIS Thorax ≥ 3 | 51 (55.4) | 9 (47.4) | 14 (48.3) | 27 (69.2) | 1 (20.0) |
| AIS Abdomen ≥ 3 | 33 (37.1) | 4 (23.5) | 13 (44.8) | 15 (39.5) | 1 (20.0) |
| AIS Extremity ≥ 3 | 30 (34.1) | 4 (25.0) | 5 (17.2) | 19 (50.0) | 2 (40.0) |
| AIS External ≥ 3 | 1 (1.1) | 0 (0.0) | 0 (0.0) | 0 (0.0) | 1 (20.0) |
| **Fluids and BP in first 24hr** |  |  |  |  |  |
| Crystalloids, L | 2.23 (1.32-3.83) | 1.53 (1.32-3.00) | 2.00 (1.10-3.30) | 2.53 (1.35-4.25) | 4.00 (3.00-6.45) |
| Red blood cells, units | 3 (1-7) | 2 (0-4) | 3 (1-4) | 5 (2-9) | 7 (0-33) |
| Massive haemorrhage | 9 (10.8) | 0 (0.0) | 1 (4.0) | 7 (18.4) | 1 (33.3) |
| Fresh frozen place, units | 2 (0-6) | 1 (0-3) | 1 (0-4) | 4 (0-8) | 11 (0-27) |
| Platelets, pools | 0 (0-1) | 0 (0-1) | 0 (0-1) | 1 (0-2) | 1 (0-3) |
| Cryoprecipitate, pools | 0 (0-2) | 0 (0-2) | 1 (0-2) | 2 (0-4) | 0 (0-11) |
| **Outcomes** |  |  |  |  |  |
| mSOFA | 56 (62.2) | 8 (47.1) | 13 (44.8) | 31 (79.5) | 4 (80.0) |
| CTCOFR, days | 9 (1-16) | 3 (1-16) | 3 (1-9) | 14 (5-16) | 16 (16-16) |
| Ventilator days | 3 (1-10) | 2 (0-11) | 2 (1-8) | 6 (2-13) | 7 (3-9) |
| Vasopressor days | 2 (0-5) | 1 (0-2) | 1.0 (0-3) | 3 (2-6) | 6 (3-7) |
| RRT | 7 (7.7) | 1 (5.6) | 0 (0.0) | 4 (10.3) | 2 (40.0) |
| Venous thromboembolism | 7 (7.6) | 0 (0.0) | 1 (3.4) | 5 (12.8) | 1 (20.0) |
| Mortality | 26 (28.3) | 5 (26.3) | 3 (10.3) | 14 (35.9) | 4 (80.0) |
| Data presented as median (interquartile range) or count (percentage).  IPF, immature platelet fraction; SBP, systolic blood pressure; INR; international normalised ratio; AIS, abbreviated injury severity score; BP, blood products; mSOFA, modified sequential organ failure assessment score; CTCOFR, Composite time to complete organ failure; RRT, renal replacement therapy. | | | | | |

| **Supplementary Table S2. Multivariable regression analysis for mortality, organ dysfunction/failure and organ failure resolution.** | | | | | | | | | | | | | | | |  |  |  | |
| --- | --- | --- | --- | --- | --- | --- | --- | --- | --- | --- | --- | --- | --- | --- | --- | --- | --- | --- | --- |
|  | **Respiratory failure** | | | **Cardiovascular dysfunction/failure** | | | **Renal dysfunction/failure** | | | **Hepatic dysfunction/failure** | | | **Mortality** | | | **CTCOFR** | | | |
|  | Adj. OR | (95% CI) | p | Adj. OR | (95% CI) | p | Adj. OR | (95% CI) | p | Adj. OR | (95% CI) | p | Adj. OR | (95% CI) | p | Adj. OR | (95% CI) | p |  |
| Sex, males | 1.57 | (1.03 to 2.40) | **0.035** | 0.61 | (0.29 to 1.32) | 0.210 | 3.57 | (2.19 to 5.83) | **<0.001** | 0.95 | (0.62 to 1.45) | 0.800 | 0.86 | (0.51 to 1.47) | 0.592 | 1.08 | (0.70 to 1.67) | 0.732 |  |
| Age, years | 1.02 | (1.01 to 1.03) | **0.002** | 1.01 | (0.99 to 1.02) | 0.466 | 1.01 | (1.00 to 1.02) | 0.130 | 0.99 | (0.98 to 1.00) | **0.048** | 1.03 | (1.02 to 1.05) | **<0.001** | 1.02 | (1.01 to 1.03) | **<0.001** |  |
| Mechanism of injury, blunt | 3.24 | (1.97 to 5.31) | **<0.001** | 2.34 | (1.21 to 4.50) | **0.011** | 0.92 | (0.56 to 1.52) | 0.750 | 1.10 | (0.68 to 1.78) | 0.699 | 1.64 | (0.77 to 3.48) | 0.199 | 2.77 | (1.64 to 4.67) | **<0.001** |  |
| Injury severity score | 1.03 | (1.01 to 1.04) | **<0.001** | 1.04 | (1.01 to 1.06) | **0.004** | 1.02 | (1.00 to 1.03) | **0.019** | 1.02 | (1.00 to 1.03) | **0.020** | 1.03 | (1.01 to 1.05) | **<0.001** | 1.06 | (1.04 to 1.07) | **<0.001** |  |
| Base deficit, mEq/L | 1.05 | (1.02 to 1.08) | **0.003** | 1.01 | (0.96 to 1.05) | 0.781 | 1.08 | (1.05 to 1.12) | **<0.001** | 0.99 | (0.96 to 1.02) | 0.652 | 1.05 | (1.01 to 1.09) | **0.006** | 1.05 | (1.02 to 1.08) | **0.004** |  |
| EXTEM <40 mm | 0.99 | (0.69 to 1.43) | 0.969 | 1.33 | (0.75 to 2.35) | 0.324 | 1.07 | (0.74 to 1.57) | 0.712 | 0.99 | (0.69 to 1.44) | 0.970 | 2.49 | (1.50 to 4.15) | **<0.001** | 1.61 | (1.10 to 2.34) | **0.013** |  |
| Total Fluids and BP in 24h, L | 1.06 | (1.00 to 1.11) | **0.035** | 1.17 | (1.05 to 1.30) | **0.005** | 1.07 | (1.02 to 1.14) | **0.011** | 1.13 | (1.07 to 1.20) | **<0.001** | 1.05 | (1.00 to 1.11) | **0.044** | 1.02 | (0.97 to 1.07) | 0.387 |  |
| Thrombocytopenia |  |  |  |  |  |  |  |  |  |  |  |  |  |  |  |  |  |  |  |
| Mild | 0.76 | (0.48 to 1.20) | 0.235 | 1.07 | (0.57 to 1.98) | 0.835 | 1.96 | (1.17 to 3.29) | **0.011** | 2.13 | (1.34 to 3.39) | **0.001** | 1.18 | (0.59 to 2.36) | 0.633 | 1.00 | (0.63 to 1.60) | 0.998 |  |
| Moderate | 1.04 | (0.63 to 1.74) | 0.871 | 1.21 | (0.56 to 2.63) | 0.621 | 2.82 | (1.61 to 4.93) | **<0.001** | 3.38 | (2.01 to 5.69) | **<0.001** | 0.73 | (0.35 to 1.53) | 0.403 | 0.97 | (0.58 to 1.62) | 0.893 |  |
| Severe | 1.09 | (0.45 to 2.64) | 0.843 | 2.15 | (0.26 to 18.1) | 0.481 | 8.33 | (2.93 to 23.6) | **<0.001** | 6.34 | (2.26 to 17.8) | **<0.001** | 0.70 | (0.23 to 2.11) | 0.529 | 2.83 | (1.07 to 7.45) | **0.036** |  |
| Mortality: R^2^ = 0.15, cases per variable = 13.8. Respiratory failure: R^2^ = 0.11, cases per variable = 38.8, survivors only. Cardiovascular dysfunction/failure: R^2^ = 0.12, cases per variable = 9.3, survivors only. Renal dysfunction/failure: R^2^ = 0.18, cases per variable = 36.9, survivors only. Hepatic dysfunction/failure: R^2^ = 0.13, cases per variable = 36.3, survivors only. CTCOFR: R^2^ = 0.18, cases per variable = 41.9. CTCOFR, composite time to complete organ failure resolution; Adj., adjusted. OR, odds ratio; CI, confidence interval; BP, blood product. | | | | | | | | | | | | | | | | | | | |

| **Supplementary Table S3. Multivariable regression analysis for organ failure resolution and organ support.** | | | | | | | | | | | | |
| --- | --- | --- | --- | --- | --- | --- | --- | --- | --- | --- | --- | --- |
|  | **CTCOFR** | | | **Ventilator days** | | | **Vasopressor days** | | | **RRT** | | |
|  | Adj. Coef. | (95% CI) | p | Adj. Coeff. | (95% CI) | p | Adj. Coeff. | (95% CI) | p | OR | (95% CI) | p |
| Sex, males | -0.17 | (-1.24 to 0.89) | 0.750 | -0.08 | (-0.41 to 0.25) | 0.616 | 0.00 | (-0.24 to 0.24) | 0.972 | 3.37 | (0.76 to 15.0) | 0.110 |
| Age, years | 0.06 | (0.03 to 0.08) | **<0.001** | 0.01 | (0.00 to 0.02) | **0.033** | 0.01 | (0.00 to 0.01) | **0.019** | 0.99 | (0.96 to 1.02) | 0.358 |
| Mechanism of injury, blunt | 2.26 | (1.09 to 3.44) | **<0.001** | 0.37 | (0.02 to 0.72) | **0.038** | 0.36 | (0.10 to 0.61) | **0.006** | 2.66 | (0.61 to 11.6) | 0.194 |
| Injury severity score | 0.15 | (0.12 to 0.19) | **<0.001** | 0.05 | (0.04 to 0.06) | **<0.001** | 0.03 | (0.02 to 0.04) | **<0.001** | 1.05 | (1.01 to 1.08) | **0.005** |
| Base deficit, mEq/L | 0.12 | (0.05 to 0.19) | **0.001** | 0.02 | (-0.00 to 0.04) | 0.094 | 0.00 | (-0.02 to 0.02) | 0.868 | 1.03 | (0.96 to 1.10) | 0.409 |
| EXTEM <40 mm | 1.44 | (0.52 to 2.36) | **0.002** | 0.19 | (-0.09 to 0.47) | 0.175 | 0.15 | (-0.05 to 0.35) | 0.149 | 0.74 | (0.26 to 2.13) | 0.575 |
| Total Fluids and BP in 24h, L | 0.07 | (-0.04 to 0.19) | 0.194 | 0.03 | (-0.01 to 0.07) | 0.175 | 0.03 | (-0.00 to 0.06) | 0.071 | 1.16 | (1.05 to 1.28) | **0.004** |
| Thrombocytopenia |  |  |  |  |  |  |  |  |  |  |  |  |
| Mild | 0.26 | (-0.89 to 1.41) | 0.655 | 0.04 | (-0.30 to 0.38) | 0.810 | -0.04 | (-0.29 to 0.20) | 0.726 | 0.76 | (0.12 to 4.84) | 0.775 |
| Moderate | 0.30 | (-0.96 to 1.55) | 0.643 | 0.09 | (-0.29 to 0.47) | 0.634 | 0.04 | (-0.23 to 0.32) | 0.757 | 1.68 | (0.31 to 9.00) | 0.547 |
| Severe | 2.27 | (0.11 to 4.44) | **0.040** | 0.85 | (0.14 to 1.55) | **0.019** | 0.70 | (0.21 to 1.19) | **0.005** | 9.22 | (1.42 to 59.8) | **0.020** |
| CTCOFR: R^2^ = 0.27, cases per variable = 85.5. Ventilator days: R^2^ = 0.19, cases per variable = 71, survivors only. Vasopressors days: R^2^ = 0.21, cases per variable = 69.5, survivors only. RRT days: R^2^ = 0.15, cases per variable = 3.3, survivors only. CTCOFR, composite time to complete organ failure resolution; RRT, renal replacement therapy; Adj., adjusted; Coef., coefficient; CI, confidence interval; OR, odds ratio; BP, blood product. | | | | | | | | | | | | |

**Supplementary Figure S1.** Haematocrit (A) and haemoglobin levels (B) over time stratified by the severity of thrombocytopenia. Bars indicate median with standard deviation.

**Supplementary Figure S2.** Immature platelet fraction (IPF) and immature platelet count (IPC) levels over time stratified by the severity of thrombocytopenia. None, no thrombocytopenia; Mild, mild thrombocytopenia; Mod, moderate thrombocytopenia; Sev, Severe thrombocytopenia. Whiskers represent 10^th^-90^th^ percentiles.

**A**

**B**

**C**

**D**

**E**

**F**

**Supplementary Figure S3.** Organ injury score trajectories stratified by the severity of thrombocytopenia. Values are mean with 95% confidence intervals.
